# Role of Zinc-Doped Bioactive Glass Encapsulated with Microspherical Gelatin in Localized Supplementation for Tissue Regeneration: A Contemporary Review

**DOI:** 10.3390/molecules26071823

**Published:** 2021-03-24

**Authors:** Dokyeong Kim, Youn-Soo Shim, So-Youn An, Myung-Jin Lee

**Affiliations:** 1 Department of Biomedicine & Health Sciences, College of Medicine, The Catholic University of Korea, Seoul 06591, Korea; dkkim2908@gmail.com; 2 Department of Dental Hygiene, Sunmoon University, Asan 31460, Korea; shim-21@hanmail.net; 3 Department of Pediatric Dentristry & Wonkwang Bone Regeneration Research Institute, College of Dentistry, Wonkwang University, Iksan-si 5453, Korea; 9543sue@hanmail.net; 4 Department of Dental Hygiene, Division of Health Science, Baekseok University, Cheonan 31065, Korea

**Keywords:** Zn-Doped Bioactive Glass (ZBG), tissue-regeneration, controlled release, gelatin microspheres

## Abstract

Gelatin, a natural polymer, provides excellent tissue compatibility for use in tissue rehabilitation. Bioactive glasses (BAG) offer superior capacity in stimulating a bioactive response but show high variability in uptake and solubility. To tackle these drawbacks, a combination of gelatin with BAG is proposed to form composites, which then offer a synergistic response. The cross-linked gelatin structure’s mechanical properties are enhanced by the incorporation of the inorganic BAG, and the rate of BAG ionic supplementation responsible for bioactivity and regenerative potential is better controlled by a protective gelatin layer. Several studies have demonstrated the cellular benefits of these composites in different forms of functional modification such as doping with zinc or incorporation of zinc such as ions directly into the BAG matrix. This review presents a comprehensive perspective on the individual characteristics of BAG and gelatin, including the synthesis and mechanism of action. Further, adaptation of the composite into various applications for bone tissue engineering is discussed and future challenges are highlighted.

## 1. Introduction

Chronic inflammatory disease leads to breakdown of the periodontal apparatus, which forms a vital component of the masticatory apparatus [1]. Conventional treatment modalities focus on mechanical procedures such as scaling and root planning, while mouth rinses are the main adjuvant method of prevention. Permanent periodic therapy and patient compliance are critical for the long-term success of such treatments. However, factors such as the host immune response are equally significant to achieve the targeted outcome.

To this end, studies have attempted to include immunomodulatory therapy in the treatment plan to supplement the host immune response. Compared with generalized oral supplementation, localized drug therapy exhibits superior efficacy, as more effective concentrations can be achieved [2]. Usually, the constant application of anti-inflammatory agents is necessary to ensure an effective concentration at the affected periodontal site. However, localized therapy often translates into the periodic use of gels or rinses, the success of which relies on patients’ compliance. Ongoing attempts to overcome these limitations have been made through research focusing on the development of micro- and nanocarriers for immunomodulatory therapies [3], such as polylactic-co-glycolic acid (PLGA), gelatin, silica, and chitosan. Local supplementation therapy has been particularly meaningful in the design of scaffolds and grafts in tissue engineering [4]. Tissue engineering has largely benefitted from the advancement of bioactive inorganic materials such as bioactive glass (BAG) or calcium phosphates, which can be modified to a different dominant element to highlight a particular response [5]. The BAG, however, possess by nature a biologically active surface and thus tends to show early degradation.

With concern to tissue regeneration and healing, the desired response is of a sustained low dose over a long period of time in contrast to an early sharp peak following a bolus dose [6]. These concerns have been mitigated to some extent by the use of gelatin coatings or encapsulation of the BAG. Thus, in the present review, we discuss the potential of BAG by underlying the mechanism of action of BAG, the important function of the local mineral ion supplementation, and the role of zinc release from BAG. Lastly, we briefly review the synthesis process and the applications outlined in the literature for gelatin-BAG combination.

## 2. Mechanism of Action of Bioactive Glass

**Figure 1:**
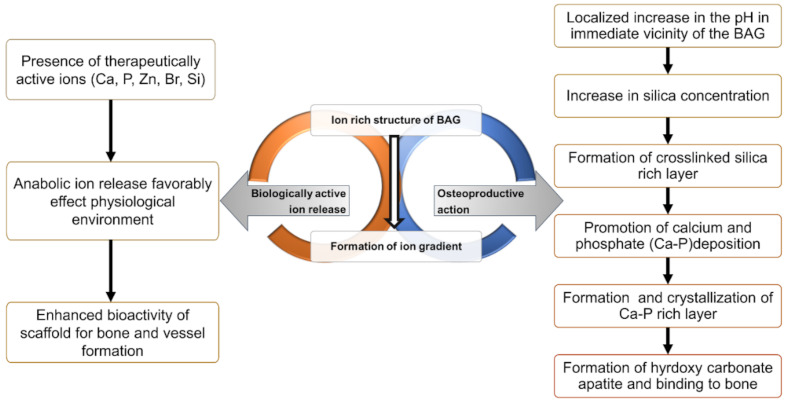
Two mechanisms of the action of bioactive glasses: facilitating growth on scaffolds by therapeutically active ion release [11] and promoting bone formation and adhesion [12].

Local host immunomodulation is used to arrest the pro-inflammatory axis of the immune response and increase the regulatory immune response [3]. Bioactive glass (BG) can act as both an inductive agent and a scaffold for tissue regrowth [7]. It is a highly biocompatible, osteoinductive and osteoconductive calcium silicate-based biomaterial [8]. BG, in addition to being osteoproductive, is an inherently restorative material. It allows bone tissue formation on the surface, bonding to surrounding living tissue when implanted in the body [9]. All BGs are functionally active due to two underlying mechanisms [10]: (i) promoting cellular adhesion and growth on the surface and (ii) stimulating biological activity such as cell growth and differentiation by the active release of component ions (Figure 1).

## 3. Use of Local Essential Mineral Supplements

Zinc is an essential mineral that plays an important role in the formation of blood vessels, bone maturation, and bacterial resistance. In animal studies by Yamaguchi et al. [13,14], the effects of zinc ions have been found to promote the binding of bone protein, calcium ion content, and increased alkaline phosphatase (ALP) activity. The concentration-dependent findings were further corroborated in the work of Ishikawa et al. [15], where an increase in the concentration of Zn caused a marked increase in cytotoxicity. These observations indicated that Zn, the sixth most abundant mineral in the body, has a significant impact on the anabolic process of bone formation, even at low concentrations.

Contemporary literature summarizes the potent effect of zinc in promoting bone development and healing by presenting evidence for the proliferation of osteoblasts and chondrocytes during endochondral bone formation. In addition, local carrier-mediated effects, such as bone cement, have also supported direct bone formation on the surface.

While the role of zinc as an essential mineral has long been known, recent research has presented the need for the future development of localized zinc-based therapy for enhanced anabolic effects. This effect of zinc ion supplementation to enhance the physiological process would improve the healing time and reduce the associated discomfort in patients with traumatic injuries of skeletal tissues [16]. To this end, zinc supplementation has been attempted through oral ingestion; however, the narrow effective dose concentration has limited this usage [17]. With the advancing development of localized micro-carrier-mediated use of Zn-doped bioactive glass (ZBG), these adverse effects can be minimized while achieving effective dose levels.

## 4. Functional Activity of ZBG

The morphological and compositional advantages of ZBG make it a multifunctional biomaterial, with both antibacterial and physiological anabolic effects such as osteogenesis and angiogenesis [18,19,20]. The antibacterial action of ZBG, similar to that of other BGs, is believed to be potent against both gram-positive and gram-negative bacteria [21]. This effect stems from the cations increasing the pH of the surroundings and from the intracellular incursion of Zn ions, causing disruption of the cell membrane [22]. The pH change also facilitates local moderation of the inflammatory reaction, and the increased resistance to microbes has shown to improve healing of both soft tissue and hard tissue wounds [23].

In addition to the antibacterial activity of Zn ions, the combined action of calcium, phosphorous, sodium, and silica ions has been shown to stimulate an anabolic response [24]. These constructive effects are dependent on the concentration of the Zn ion released, which has been shown in previous studies to be effective at low concentrations and exponentially inhibitory with the increase of the zinc oxide content of the glass [20,25].

However, its ability to undergo resorption limits the duration of action of the glass particles, requiring repeated supplementation [26]. Next, we discuss the application of a biodegradable scaffold that can control the rate of release, or in this case, slow the resorption process to augment the duration of the action of BG.

## 5. Role of Micro/Nanocarriers: Gelatin Microspheres

The prerequisite for a long-term effect is controlled release, which is made possible by encapsulation. In this procedure, a scaffold suitable to carry the active agent is prepared as batches of micro- or nanosized enveloped particles [27]. Gelatin has been proposed as a favorable encapsulating material for localized delivery of immunomodulatory agents. Gelatin is a proteinaceous biodegradable polymer obtained from the partial hydrolysis of collagen from the skin, connective tissues, and bones of animals. Biocompatible gelatin is commonly used in tissue engineering studies to support the adhesion, proliferation, and differentiation of cells. The favorability for use of gelatin stems from the fact that the naturally derived polymer is cost effective, biocompatible, relatively easy to manipulate, and can form stable thin coatings [28,29]. At the same time, it is vexed with inherently low consistency in dissolution and poor mechanical integrity [30,31].

To augment the physical characteristics and improve stability, the gelatin microspheres, which act as carriers or scaffolds, are typically prepared by crosslinking. Crosslinking is a process by which a bond, physical or chemical, in nature can be formed between the active functional groups of a polymer chain, such that a stable network can be formed. To this end, a commonly used chemical crosslinking agent, glutaraldehyde is used, which improves the stability of the gelatin molecular structure [32]. Gelatin is dissolved in water with glutaraldehyde in an oil emulsion using the coacervation phase separation method. The effect of various parameters such as temperature, pH, degree of crosslinking, and the amount of gelatin on the release kinetics from gelatin microvesicles and nanocarriers have been extensively studied [33].

## 6. Synthesis of Gelatin-ZBG Microcapsules

In addition to cross linkage, a gelatin structure also benefits from the inclusion of an inorganic material. In other words, a synergistic effect can occur by formation of composite with glass, such as of ZBG with gelatin [12]. ZBG is commonly synthesized using melt-mixing or sol-gel cycling after milling to obtain nanosized active particles [34]. The composition of the glass and the method of fabrication determine the density and structure of the particles. The glass particles can be used as scaffolds for nanoparticles while inherently acting as active agents.

Gelatin micro- and nanoparticles can be obtained by many methods such as solvent evaporation, monomer polymerization, salting out procedure, emulsification, reverse phase preparation, coacervation, and sonochemical methods [35]. All of the above methods carry their respective details; however, we will briefly elaborate the differences with their core principle to draw a simple distinction. The solvent evaporation and emulsification procedures are based on the same principle, which is the single water-in-oil emulsion technique. The solvent evaporation differs from the emulsion technique as it starts with a polymeric phase while the emulsion technique involves monomers reacting during synthesis. In addition, it also includes a high speed homogenization and complete desiccation of the thus obtained particles in a powder state, which are directly affected by the stirring speed and homogenization method [36]. Furthermore, the basic emulsion technique can differ with the presence of a surfactant, and the type and rate of polymerization. The salting out process is also similar to the emulsion solvent method coupled with the principle of the salting-out process, where the addition of the salting-out agent aqueous solution is added at a constant stirring. It is then followed by a dilution of water and cross flow filtration to obtain polymer particles [37]. The sonochemical method is another commonly employed method where capsulation of gelatin is obtained by deploying an ultrasonic horn at the interface of air and gelatin, resulting in acoustic cavitation and particulate breakdown in size [35]. Barring the sonochemical method, other processes result in the synthesis of a solid particle with or without a coating rather than encapsulation, per se.

**Figure 2:**
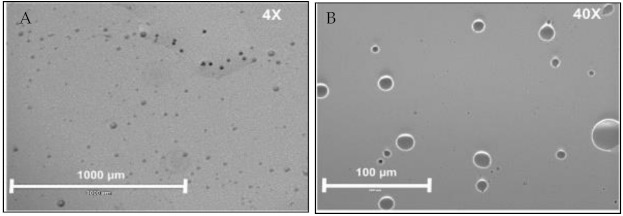
Light microscopy images of the gelatin microspheres.

**Figure 3:**
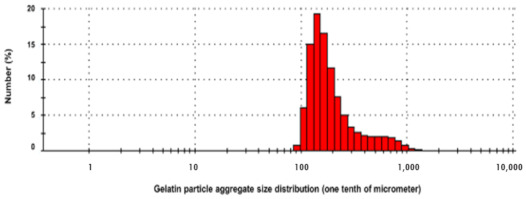
Size distribution of the gelatin microparticles.

In addition to the core synthesis procedure, thermal treatment of the formed microspheres also influences the release of the active chemical. The thermal process, which leads to hardening, can be broadly specified as in-situ (during microsphere production) and post-production methods (thermal drying after isolation) [38]. Among the various methods deployed, emulsification with controlled crosslinking offers a reproducible procedure for the preparation of gelatin-ZBG microspheres. To briefly elaborate, gelatin-ZBG microspheres are prepared by the phase separation method utilizing temperature change as follows: A gelatin solution is prepared by adding 2 g of gelatin to 10 mL of distilled water preheated to 50 °C. Subsequently, ZBG is added to the solution at the same temperature while continuously stirring. After dispersion, the solution is poured drop-wise into the oil phase (50 mL olive oil at 50 °C) [39]. While stirring, the flask is cooled using an ice bath. After 30 min, 50 mL of chilled acetone (4 °C) is added. After 1 h of stirring, microspheres are collected by filtration using 200-micron filter paper and then washed twice with acetone (500 mL) to remove residual olive oil, followed by repeated washing with 70% ethanol to completely remove the oil [40]. The final wash is done with deionized water, and beads are dried at room temperature and then at 4 °C for 48 h. The synthesized microspheres, when imaged by light microscopy, can be seen in Figure 2. The size distribution, evaluated with dynamic light scattering (DLS), shows a high density of microscopic particles, as shown in Figure 3.

The composite of gelatin and BAG can exhibit variations in the physical properties with changes in the proportion of inorganic constituents (mostly BAG) interacting with gelatin matrix. Mozafari et al. detailed the chemical interaction between the gelatin and the bioactive glass for 3D nanocomposite scaffolds [41] and the microporous scaffolds [30]. Briefly, the gelatin molecules form a complex with the calcium ions from the BAG. It progresses with assembly of negatively charged phosphate ions and the maturation of the complex by the formation of chemical bonds between phosphate ions with carboxyl and amide groups of gelatin molecule. The different methods of composite adaptation in scaffold and hydrogel formation for tissue-engineering, such as freeze drying, solvent casting, crosslinking gelation, electrospinning and solid free fabrication were reviewed in detail with their respective properties by Sergi et al. [42].

## 7. BAG Gelatin Combination in Tissue Engineering

Scaffold based application of the gelatin/BAG for bone tissue engineering were reported in detail by Mozafari et al. [30]. The findings of their paper are particularly encouraging, as they successfully reported the development of a chemical bonding between the BAG nanoparticles and gelatin structure. BAG-gelatin composite was fabricated by layer casting and lyophilization with glutaraldehyde cross-linker. The formation of the chemical bonding was reported to take place in three steps involving BAG calcium (Ca^2+^) ions with gelatin molecules, assembly of phosphate ions on the Ca^2+^/Gelatin complex and lastly the formation of bonds between the organic molecular groups. This sequence of reaction provided integrity to the nanocomposite. In addition, electrostatic and stabilization enhanced the stability of the gelatin molecules to prevent agglomeration. With their findings of enhanced physical characteristics, biocompatibility, and osteogenicity, they proposed complexing BAG/gelatin in bone tissue engineering applications.

Maximizing the synergistic use of a composite structure, multiple studies have incorporated ceramics in various forms such as hydroxyapatite, tricalcium phosphate, and BAG, with the commonality of using lyophilization in fabricating the composite structure. The composite structure again shows variation in its features with differences in pore structure, and for that reason a homogeneity in pore structure is believed to be of superior characteristics. To this end, J. Lacroix et al. [43] proposed the use of a PolyMethylMethAcrylate (PMMA) microsphere as a porogen to obtain a good composite structure of BAG/gelatin. The methodology allows for a controlled and uniform pore structure formation, which can enhance the mechanical properties while allowing versatility in the pore structure.

For localized tissue engineering applications, ion supplementation from dissolution of BAG aids in improving the local tissue response. In addition, the crosslinking capability of the gelatin can permit a chemical bond with local antibiotic chemicals. This characteristic in bone tissue engineering was reported in the study by Li et al. [32] by combining the polyguanidine into the gelatin layer, which then encapsulates the BAG. Their study reported a long-term durability of the gelatin films over BAG, lasting over 10 days. The polyguanidine had effective anti-bacterial action against both gram positive and negative bacteria while the composite structure maintained its biocompatibility with osteogenic cells. These findings were encouraging for the exploration of functionalized composite of gelatin with BAG.

Another possibility in augmentation of the BAG/gelatin scaffold was explored by Johari et al. [44] where they modified the nanocomposite of Bag/gelatin by seeding with osteoblast cell lines, mimicking a three-dimensional cell culture model. A sterile scaffold of nanocomposite fabricated by layer casting was seeded with osteoblast suspension and then cultured. The MTT assay results showed good cellular compatibility and the histological analysis confirmed potential to bone formation and repair of the defect. This study also highlighted the role played by local ionic supplementation, which not only adds to resistance from infection but also helps in promoting growth of the osteogenic cells.

Zheng et al. [45] also discussed the advantages of functionalizing the composite of BAG with gelatin. While the established merits of BAG with gelatin composite of good mechanical properties can be achieved with higher reproducibility, the magnitude of bioactivity (the ability to form an apatite-like structure) is variable. In their research, Zheng et al. reported the functionalization of glass with copper. This differs from the cell seeding mentioned above, as it is only limited to the surface of the composite structure and is not part of the 3D network. On similar grounds, Guo et al. [46] explored the effects of the bioactive glass/gelatin/ZnO composite used in fabrication of scaffolds. Their observations were derived from the experiments evaluating the mechanical and physical properties of the nanocomposite of BAG/gelatin, benefitting from the tetrapodal structure of the ZnO. A 2 wt % of the ZnO addition to the nanocomposite showed the highest improvement in the strength and modulus of the fabricated scaffolds. In addition, they also observed a relatively increased proliferation in the mesenchymal cell activity. Covarrubias et al. [9] used a chitosan–gelatin polymer blend and the nanoparticles of BAG for the fabrication of scaffolds. The scaffolds presented with good cytocompatibility and bioactivity in both in vitro and in vivo experiments with excellent new bone formation capacity. The results of this study were based on a hypothesis aimed at maximizing the effect of Zn on anabolic protein metabolism. To this end, Raz et al. [47] reported a method for the synthesis of Zn containing BAG and for preparing the composite with gelatin to synthesize the scaffold in bone tissue engineering. They reported a combination of bioactivity, cytocompatibility and improved pore structure, which was deemed suitable to promote osteoblastic growth.

Moreira et al. [48] presented an alternative method for the use of gelatin/BAG nanocomposite as hydrogels. They reported the characteristics of thermosensitive hydrogels based on gelatin/BAG nanoparticles with their in vitro and in vivo behavior. The cells that seeded injectable hydrogels with a chitosan/gelatin/BAG composite showed excellent cell compatibility with no adverse reaction in animal studies. In addition, they reported a notable increase in cell growth of more than 20% for the differentiation of the mesenchymal stem cells. This was also concordant with the improvement in the bone tissue volume they reported via computed tomography experiment from the rat model.

## 8. Summary and Future Perspectives

ZBGs have shown promising developments in recent years; they have positive effects on wound healing by accelerating the return to hemostasis, reducing localized inflammation, and stimulating the anabolic activities of epithelial and osteoblast lineage cells in soft and hard tissue. These effects are maximized with controlled delivery, thus pushing the boundaries of biomedical research to develop a functional and accessible modality for the application of ZBG.

The papers reported in this review highlight the basics of gelatin/BAG composite, their individual characteristics, and the advantages of combining them as a composite. The versatility of size gives options in adapting the gelatin/BAG composite into various forms for bone tissue regeneration. The functionalization of BAG or the polymeric gelatin increases the range of application that can be observed in the in vivo experiment reported. A better understanding of how specific properties of BAG/gelatin composites affect cellular behavior is essential to understand for the fabrication of a tissue-engineering substitute with specific biological responses. The major challenges moving forward will be the optimization of the biological performance with the inherent characteristics of gelatin and bioactive glass [42].

Adaptation of localized supplementation procedures is of increasing demand in both medical and dental therapeutics. While the majority of tissue regeneration research focuses on the promotion and rehabilitation of bone, the actively driven research of human pulpal stem cells can also benefit from adapting the 3D cellular culture process using gelatin/BAG composite.

## Data Availability

Not applicable.
